# Quality assurance of craniospinal irradiation in helical tomotherapy using a dose reconstruction tool based on leaf open time

**DOI:** 10.1002/acm2.70139

**Published:** 2025-07-13

**Authors:** Xiunan Wang, Wenjie Ni, Yongqing Ge, Bo Liu, Qin Wang, Linan Song, Hui Yang, Yizhen Jin, Xiaofeng Mu

**Affiliations:** ^1^ Department of Radiation Oncology Beijing Shijitan Hospital of Capital Medical University Beijing China

**Keywords:** craniospinal irradiation, dose reconstruction, helical tomotherapy, leaf open time

## Abstract

**Objective:**

The delivery quality assurance (DQA) of craniospinal irradiation (CSI) due to the target length results in no ideal verification devices. Delivery Analysis (DA) could calculate the dose distribution based on the measured multi leaf open time in helical tomotherapy (HT). This study aimed to evaluate the efficacy of DA for DQA of CSI in HT.

**Material and Methods:**

32 CSI plans were classified into two groups based on γ analysis of the PTV‐cranial and PTV‐spine plans using a 2D ionization chamber matrix (MatriXX). Plans with γ passing rates ≥ 95% at 3%/2 mm were classified as the passed group, while those < 95% were classified as the failed group. Receiver operating characteristic (ROC) curves identified optimal passing rate threshold for DA in HT. Logistic regression analyzed risk factors for DQA failure, and failed plans were reoptimized according to the adjusted parameter.

**Results:**

For PTV‐cranial plans, 30 passed and two failed; for PTV‐spine plans, 21 passed and 11 failed. ROC analysis revealed areas under the curve of 0.858 (PTV‐cranial, threshold: 89.0%) and 0.714 (PTV‐spine, threshold: 86.0%). Logistic regression identified planned modulation factor (MF‐plan; *p *= 0.046; *p *= 0.023) and actual modulation factor (MF‐actual; *p *= 0.027; *p *= 0.008) as independent risk factors for DQA failure in both MatriXX and DA. Additionally, beam on time (*p *= 0.043), gantry period (*p *= 0.007) and maximum leaf open time (*p *= 0.007) were identified as independent risk factors for DA. Reoptimization of failed plans with MF‐plan = 2.6 significantly improved passing rates in DA (73.70% ± 13.30% vs. 88.20% ± 12.30%; *p *= 0.010) and MatriXX (91.20% ± 2.60% vs. 96.10% ± 1.40%; *p* < 0.001).

**Conclusion:**

Delivery Analysis could be a feasible tool for DQA of CSI in HT. Increasing the MF‐plan is recommended to enhance the passing rate.

**Abbreviations**
AAPM: American Association of Physicists in Medicine
CI: confidence interval
CSI: craniospinal irradiation
CT: computed tomography
DA: delivery analysis
DQA: delivery quality assurance
DVH: dose‐volume histograms
HT: helical tomotherapy
LOT: leaf open time
MatriXX 2D: ionization chamber matrix
MF‐plan: The planned modulation factor
MF‐actual: actual modulation factor
MF: modulation factor
MU: monitor unit
MVCT: megavoltage cone beam computed tomography
OARs: organs at risk
OR: odds ratio
ROC: receiver operating characteristic
SD: standard deviation
SIOPE: European Society for Pediatric Oncology
TG: task group

## 1 INTRODUCTION

Craniospinal irradiation (CSI) is essential for medulloblastoma, germ cell tumors, and leukemia with central nervous system involvement that metastasize along the subarachnoid space.^1^, ^2^, ^3^ The extended radiation field poses a challenge because traditional techniques, such as three‐dimensional conformal radiotherapy, intensity modulated radiotherapy, and volumetric modulated arc therapy, require multiple isocenters. Gram et al.^4^ reported weak to moderate correlations between setup uncertainties and factors such as the number of isocenters, patient age, and the use of anesthesia. It is important to note that there could be variations in the relative distance between isocenters (Linac patients with multiple isocenters) which can lead to large differences between the expected and actual dose distribution. Helical tomotherapy (HT) could achieve single‐isocenter irradiation, superior target conformity, uniformity, and organ protection.^5^, ^6^, ^7^ Delivery quality assurance (DQA) before treatment is essential and important. Commercial verification devices are typically restricted by size (20–27 cm). Therefore, the CSI plan must be validated at least twice. Lee et al.^8^ utilized a 0.6‐cm^3^ Farmer ionization chamber, 2D diode detector (MapCHECK), and 3D diode detector (ArcCHECK) to verify the CSI dose, but ionization chambers are limited to point dose information, MapCHECK can only be placed horizontally with nominal gantry (0°/180°) for overlap region to avoid the device electronics, and ArcCHECK can only be used to verify the dose of each isocenter separately. Misa et al.^9^ used fluence maps of electronic portal imaging device combined via scripting for dose verification, but the methodology lacked cross‐verification and was exclusive to Varian accelerators. Da et al.^10^ employed optical computed tomography (CT) and radiochromic gel dosimeters for 3D dose measurements, yet measurement length and cost remained problematic. Thus, current detectors and methods are insufficient for single‐session DQA of CSI.

Delivery Analysis (DA) is an independent workstation software capable of reconstructing 3D dose distributions on CT scans using LOT measured by the HT detector array, allowing comparison between the original and reconstructed doses. DA only needs to be validated once before treatment, and its operation is simple. Several studies have validated the reliability of reconstructing 3D dose distributions using LOT in conventional irradiation techniques.^11^, ^12^ DA's precision detectors and advanced reconstruction algorithms ensure dose reconstruction accuracy, making it particularly suitable for DQA of CSI. This study aimed to evaluate the feasibility and effectiveness of using DA for CSI in HT, analyze the risk factors affecting the passing rate, and provide a reference for clinical DQA.

## 2 MATERIALS AND METHODS

### 2.1 Database

The study included 32 patients (males = 23, females = 9; aged between 6 and 42 years, median = 10 year) who received CSI between September 2022 and March 2024. There were 22 cases of leukemia with central nervous system involvement, seven cases of medulloblastoma, and three cases of germ cell tumors. The target was delineated according to the European Society for Pediatric Oncology (SIOPE) consensus guidelines,^13^ which include PTV‐cranial and PTV‐spine. A dose of 1.5–2.0 Gy per fraction was delivered. The planning was done using Precision (v1.1.1.1, Accuray, Inc., USA), with a uniform field width of 5 cm. Other planning parameters prioritized plan quality, rotation period, and beam on time (Table 1).

**TABLE 1:** Patient's plan parameters.

	PTV‐spine				PTV‐cranial			
	Group passed (Average ± SD)	Group failed (Average ± SD)	t	*p*‐value	Group passed (Average ± SD)	Group failed (Average ± SD)	t	*p*‐value
Total dose (Gy)^*^	18 (9–18)	18 (17–36)	−0.949	0.343	18 (11.5–24)	36 (36–36)	−1.862	0.063
Fraction dose (Gy)^*^	2 (2–2)	2 (1.8–2)	−1.051	0.293	2 (2–2)	2 (2–2)	0.687	0.492
Fractions^*^	9 (4.5–10)	10 (9–18)	−1.363	0.173	9 (5.75–12)	18 (18–18)	−1.836	0.066
Total MU	86 575.97 ± 42 342.13	104 882.30 ± 46 949.16	1.120	0.272	89 748.76 ± 43742.14	139 653.90 ± 12691.72	−1.587	0.123
Fraction MU	9227.69 ± 2860.33	8603.33 ± 1868.27	−0.652	0.519	8440.04 (7767.29‐10486.97)^*^	7758.55	−1.522	0.128
Pitch^*^	0.444 (0.43‐0.444)	0.444 (0.430‐0.444)	−0.685	0.494	0.444 (0.43‐0.444)	0.43 (0.43‐0.43)	1.469	0.152
Plan MF^*^	2.4 (2.05‐2.55)	2 (2‐2)	−1.916	0.055	2.2 (2.0‐2.5)	2.00 (2.00‐2.00)	−1.680	0.093
Actual MF	2.07 ± 0.22	1.9 ± 0.16	−2.281	**0.030**	2.03 ± 0.21	1.80 ± 0	1.469	**<0.001**
Beam on time (s)	685.25 ± 162.11	603.04 ± 130.96	−1.449	0.158	607.45 (551.50 ± 770.425)	543.85	−1.090	0.276
Maximum LOT (s)	375.99 ± 48.27	351.15 ± 53.57	−1.333	0.193	368.94 ± 52.09	345.20 ± 5.52	0.635	0.531
Mean LOT (s)	181.59 ± 15.12	183.48 ± 17.31	0.321	0.751	181.41 ± 15.83	194.60 ± 1.27	−1.16	0.255
SD LOT (s)	75.65 ± 13.09	79.51 ± 15.62	0.741	0.465	76.35 ± 14.13	86.35 ± 1.91	−0.985	0.332
Volume (cm^3^)	448.48 ± 175.01	410.78 ± 60.68	−0.890	0.381	1932.98 ± 210.56	1744.87 ± 25.21	1.244	0.223
Gantry period (s)	19.22 ± 2.46	17.96 ± 2.72	−1.328	0.194	18.85 (17‐20.50)^*^	17.70	−0.467	0.640
Rotations	35.5 ± 6.78	34.25 ± 10.23	−0.415	0.681	33 (29.38‐38.08)^*^	30.70	−0.779	0.436
Length (cm)	520.08 ± 109.31	495.12 ± 80.58	−0.666	0.510	162.46 ± 13.87	147.50 ± 9.19	1.49	0.147
Couch travel distance (cm)	771.52 ± 135.23	694.79 ± 83.83	−1.710	0.098	719 (656.68‐834.03)^*^	666.00	−0.856	0.392

Abbreviations: LOT, leaf open time.; MF, modulation factor; MU, monitor unit; SD, standard deviation.

*: Non‐normal distribution were described as medians with their ranges.

### 2.2 MatriXX for DQA evaluation

MatriXX (IBA, Schwarzenbruck, Germany), is a 2D air ionization chamber matrix with 1,020 chambers arranged in a 32 × 32 cm matrix. Each chamber has a sensitivity volume of 0.07 cm^3^, with a spacing of 0.762 cm between chambers, providing an effective detection range of 24 × 24 cm. When used with the Miniphantom (IBA, Schwarzenbruck, Germany; electron density of 1.014), it is suitable for DQA in HT. Instead of validating the CSI target using an ionization chamber alone, we verified the plan after calibrating the absolute dose to MatriXX by ionization chambers. Therefore, we could obtain absolute dose distribution and simplify the verification process. MatriXX calibration involved designing a tomotherapy pre‐irradiation program (gantry = 0°; field size = 5 × 40 cm) for 300 cGy pre‐irradiation to ensure dose response consistency, followed by a calibration program (gantry = 0°; field size = 5 cm × 20 cm; source to surface distance = 85 cm; equivalent water thickness = 6.8 cm) with a static couch. Ionization chamber measurements using a uniform water phantom were input into MatriXX software for absolute dose calibration. The 32 CSI plans were then transferred to the MatriXX virtual phantom for calculation, verifying the PTV‐cranial and PTV‐spine segments separately.^14^ For measurements intended to evaluate planned dose accuracy, the comparison criteria would ideally be based on clinical organ‐by‐organ tolerances. For example, the tumor dose tolerance specification might be 3%, and the spatial accuracy requirement might be 2 mm at the edge of the spinal cord. Therefore, 2D γ analysis in absolute dose mode was conducted with a 3%/2 mm standard recommended by American Association of Physicists in Medicine (AAPM) Task Group (TG)‐218. We set the dose threshold to 30% for MatriXX which is almost consistent with DA's validation range. Plans with γ passing rates ≥ 95% were considered as passed; otherwise, they were considered failed.

### 2.3 DA for DQA evaluation

The megavoltage cone beam computed tomography (MVCT) is an arc‐shaped detector array, which installed on the gantry's slip‐ring opposite to the linear accelerator. It consists of 640 dual‐channel ionization chambers filled with xenon gas. During the irradiation, the detector analyzed the pulse‐by‐pulse data and measured the individual LOTs using the high‐signal pulses for each delivered projection. Post‐irradiation, DA reconstructed the 3D dose distribution on the patient's CT scans using MLC‐LOTs sinogram, and evaluated it through 3D γ analysis and dose‐volume histograms (DVH). To ensure accuracy, we recalibrated the HT output dose and reset the signal threshold in the algorithm according to DA manual calibration data. Validation plans for the 32 CSI clinical plans were generated using the Precision planning system, with no phantom on the treatment couch during irradiation. 3D γ analysis followed the 3%/2 mm criteria recommended by AAPM TG‐218. There is no dose threshold in DA, which analyzes all voxels of the given structure.

### 2.4 Reoptimization of the failed plans

Validation failed PTV‐spine plans were reoptimized by increasing the MF‐plan to 2.6. Other parameters remained unchanged. The new plans had to meet clinical requirements for target coverage and the organ at risk. Dose validation was conducted using both the DA and MatriXX.

### 2.5 Statistical analysis

Independent samples *t*‐test was used for normally distributed data, paired samples *t*‐test was used for paired samples, and Mann–Whitney *U* test was used for non‐normally distributed data. Receiver operating characteristic (ROC) curves identified optimal passing rate thresholds of DA‐reconstructed 3D doses. Logistic regression was used to identify risk factors affecting the passing rate. Correlation analysis of risk factors used Spearman's correlation analysis. Dose validation of reoptimized plans used paired samples *t*‐test. Statistical analysis was conducted using IBM SPSS Statistics for Windows, Version 20.0 (IBM Corp., Armonk, NY, USA), with *p* < 0.05 considered significant.

## 3 RESULTS

### 3.1 DQA results

Among the 32 PTV‐cranial plans, 30 passed, and two failed; among the 32 PTV‐spine plans, 21 passed, and 11 failed. For PTV‐cranial plans, the γ passing rate was 97.50% ± 1.90% for MatriXX and 92.60% ± 9.90% for DA. The respective values for PTV‐spine plans were 94.90% ± 3.30% and 79.90% ± 15.40%.

### 3.2 Optimal passing rate threshold of DA‐reconstructed 3D Dose

**FIGURE 1:**
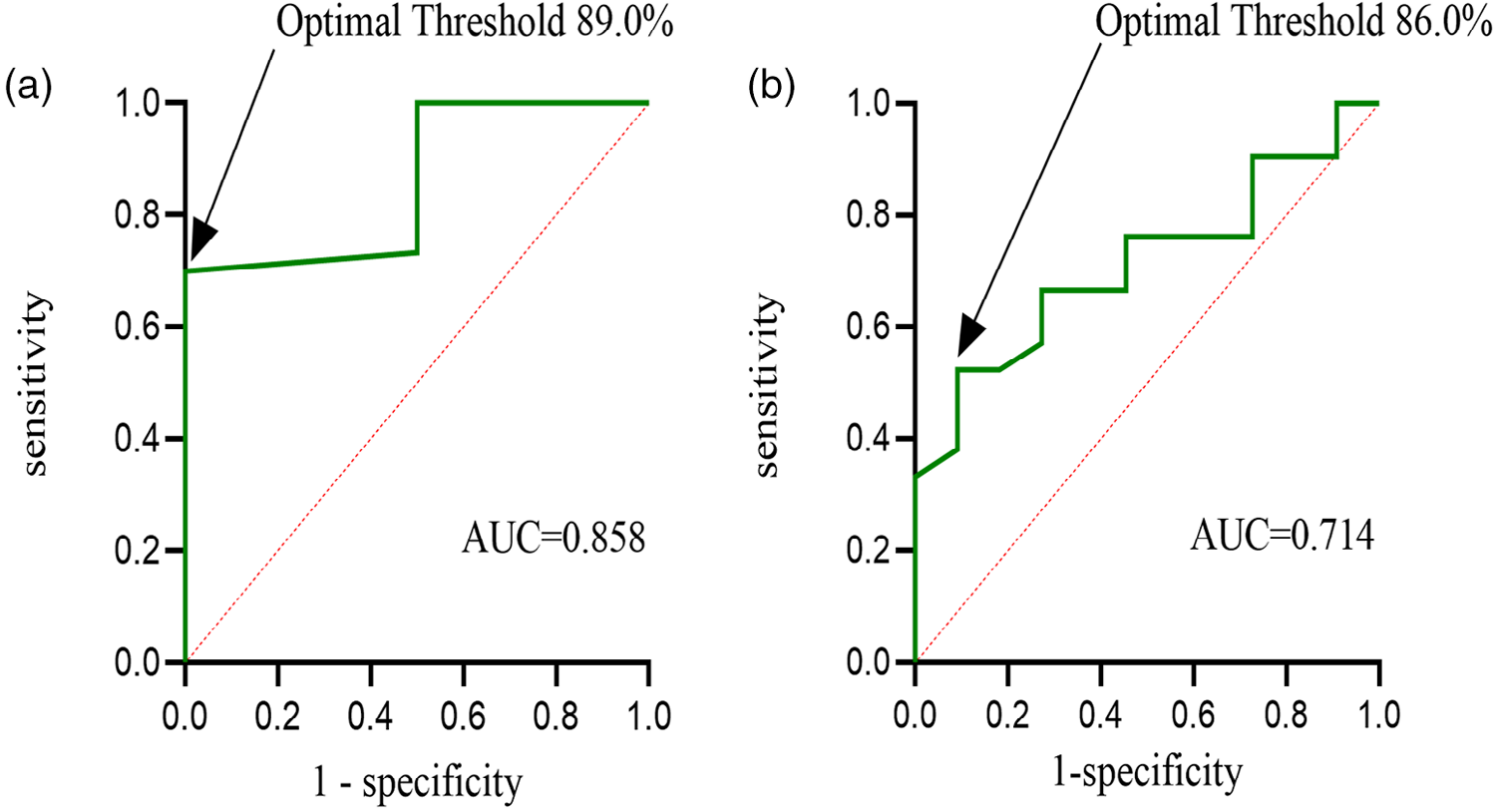
ROC curves for DA‐reconstructed 3D doses. (a) PTV‐cranial; (b) PTV‐spine.

Figure 1 shows the ROC curves for the γ passing rate of DA‐reconstructed 3D doses. The area under the curves for the DA‐reconstructed 3D dose was 0.858 in the PTV‐cranial plan and 0.714 in the PTV‐spine plan. The optimal passing rate thresholds were 89.0% for the PTV‐cranial plan and 86.0% for the PTV‐spine plan. The sensitivity was 0.700 for the PTV‐cranial plan and 0.524 for the PTV‐spine plan. The specificity was 1.000 for the PTV‐cranial plan and 0.900 for the PTV‐spine plan.

### 3.3 Predictors of DQA Failure

Among the 32 PTV‐cranial plans, only two failed verification, with γ passing rates of 92.75% and 90.10%, both higher than the 3%/2 mm criteria action limits recommended by AAPM TG‐218 (< 90%). Therefore, logistic regression analysis was only conducted for the PTV‐spine plans. The results showed that the MF‐plan (*p *= 0.046; *p *= 0.023) and MF‐actual (*p *= 0.027; *p *= 0.008) were independent risk factors for DQA failure for both MatriXX and DA; Beam on time (*p *= 0.043), gantry period (*p *= 0.007) and Maximum LOT (*p *= 0.007) were independent risk factors for DA (Table 2). Significant positive correlations were observed between MF‐plan and MF‐actual (*R* = 0.939, *p* < 0.001), MF‐plan and beam on time (*R *= 0.380, *p* = 0.032), MF‐plan and Maximum LOT (*R *= 0.703, *p* < 0.001), and MF‐plan and gantry period (*R *= 0.703, *p* < 0.001). Therefore, multivariate logistic regression was not conducted for the these parameters.

**TABLE 2:** Univariate logistic regression analysis of risk factors affecting DQA failure.

	MatriXX			DA		
Treatment planning parameters	OR	95%*CI*	*p*‐value	OR	95%*CI*	*p*‐value
Total dose	1.001	1.000–1.001	0.160	1.000	1.000–1.001	0.469
Fraction dose	0.954	0.891–1.022	0.180	0.948	0.858–1.047	0.294
Fractions	1.118	0.966–1.295	0.135	1.062	0.918–1.228	0.416
Total MU	1.000	1.000–1.000	0.230	1.000	1.000–1.000	0.750
Fraction MU	1.000	1.000–1.000	0.593	1.000	1.000–1.000	0.464
Pitch(0.43 vs. = 0.444)	1.591	0.356–7.112	0.543	0.341	0.070–1.654	0.182
Plan MF(≥2.3 vs. <2.3)	6.000	1.033–34.844	**0.046**	6.667	1.306–34.027	**0.023**
Actual MF (>2.0 vs. ≤2.0)	7.312	1.249–42.813	**0.027**	11.250	1.858–68.132	**0.008**
Beam on time	0.996	0.990–1.002	0.195	0.995	0.989–1.000	**0.043**
Maximum LOT	0.990	0.973–1.006	0.217	0.969	0.946–0.992	**0.007**
Mean LOT	1.013	0.963–1.064	0.622	0.983	0.936—‐1.033	0.500
SD LOT	1.035	0.973–1.101	0.279	1.024	0.968–1.084	0.412
Volume	0.998	0.992–1.004	0.487	0.995	0.988–1.000	0.052
Gantry period	0.833	0.601–1.156	0.275	0.532	0.336–0.844	**0.007**
Rotations	0.983	0.891–1.085	0.739	0.990	0.904–1.085	0.836
Length	0.998	0.990–1.006	0.597	0.995	0.988–1.003	0.236
Couch travel distance	0.993	0.984–1.002	0.123	0.998	0.992–1.004	0.572

Abbreviations: CI, confidence interval; LOT, leaf open time; MF, modulation factor; MU, monitor unit; OR, odds ratio; SD, standard deviation.

### 3.4 Reptimization for the failed plans

**FIGURE 2:**
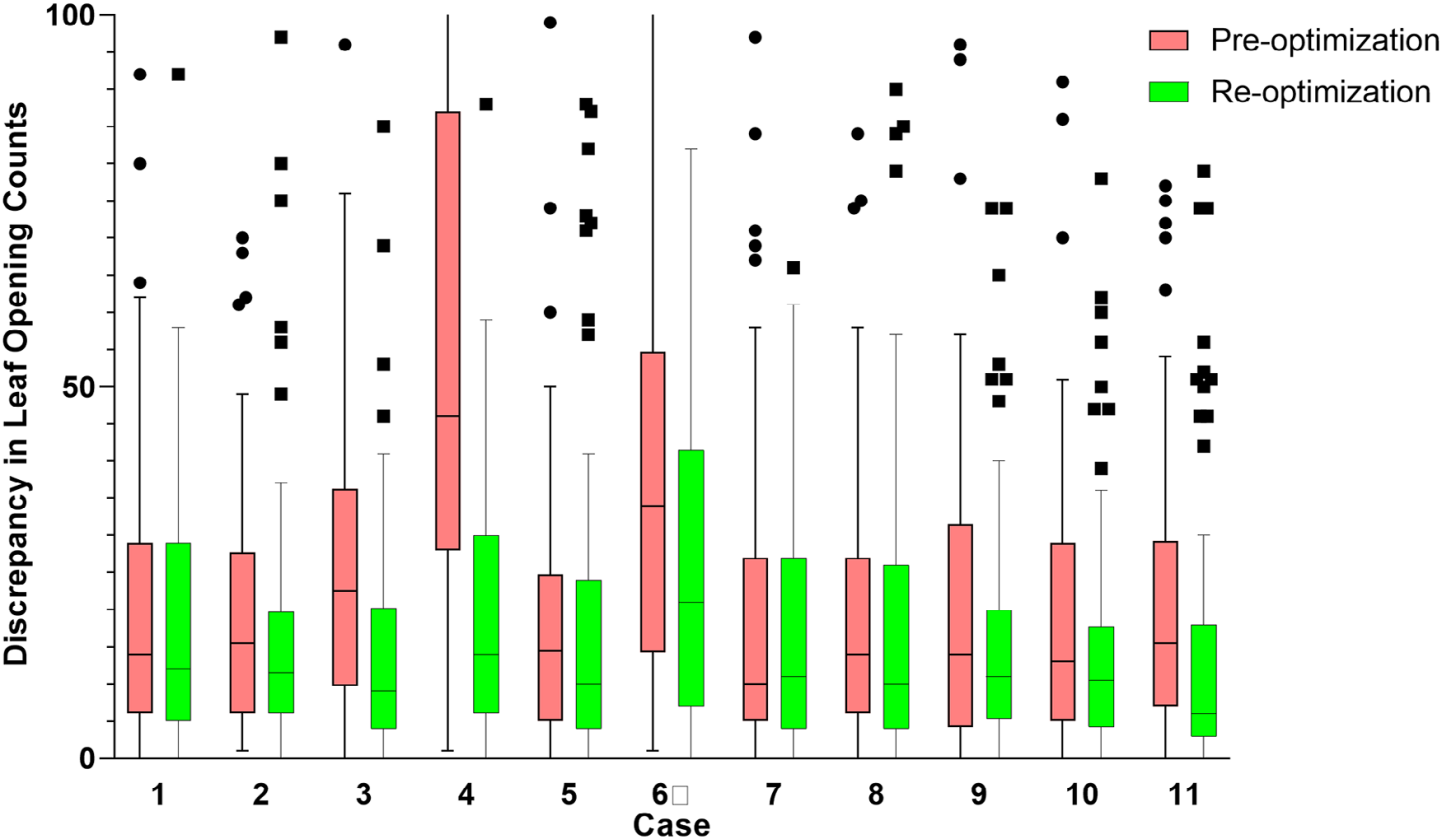
The discrepancy in leaf openings counts of 11 failed plans was reduced after optimization (Pre‐optimization: revised histogram of leaf opening counts between planned and reconstructed distributions before optimization; Re‐optimization: revised histogram of leaf opening counts between planned and reconstructed distributions after optimization).

**FIGURE 3:**
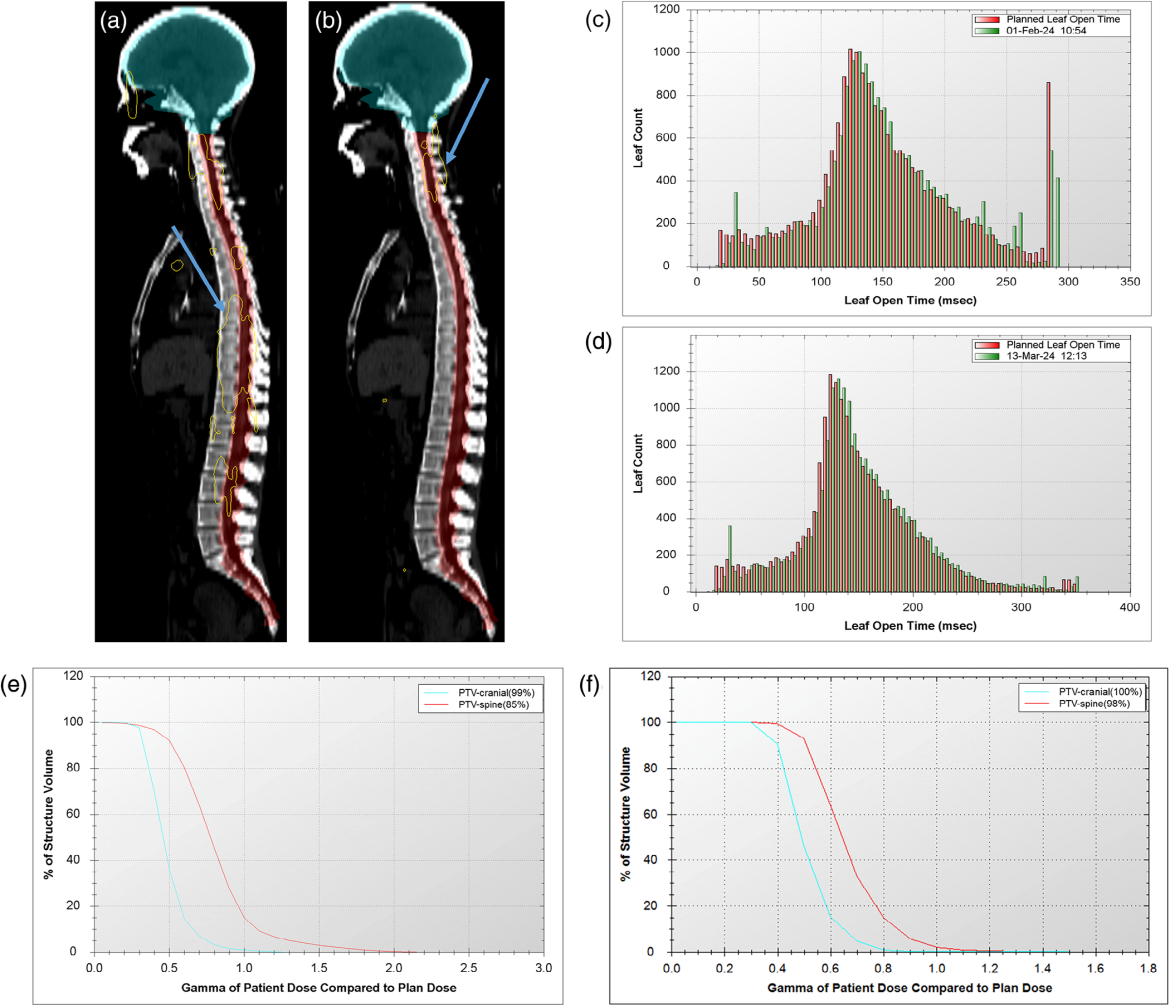
Comparison of plan verification before and after optimization for one patient. The yellow dose line indicated by blue arrows represents the distribution of dose deviation greater than 3% at the 3%/2 mm criterion before (a) and after (b) optimization. The red and green labels represent the planned and actual leaf open time in LOT histograms before (c) and after (d) optimization. The percentage of cumulative gamma histograms represent the γ passing rate before (e) and after (f) optimization.

Since an MF‐plan < 2.3 was identified as a risk factor for DQA failure, we calculated the average MF‐plan for plans with MF‐plan ≥ 2.3, yielding a rounded average of 2.6. Reoptimizing the 11 failed PTV‐spine plans using a new MF‐plan of 2.6 improved the DA (73.70% ± 13.30% vs. 88.30% ± 12.39%; *p *= 0.010) and MatriXX (91.20% ± 2.60% vs. 96.10% ± 1.50%; *p* < 0.001) passing rates. After optimization, the deviation between the reconstructed and planned LOTs decreased (Figure 2). Statistical analysis showed that the proportion of LOTs exceeding 235 ms decreased after optimization (23.40% ± 7.90% vs. 19.90% ± 1.80%; *p *= 0.023). Figure 3 showed one of the 11 patients, the 3D dose deviation between the planned and reconstructed plans was significantly reduced after optimization (Figure 3a, b). The decline was particularly notable in regions with a long LOT (Figure 3c, d). Volumes that failed verification decreased significantly in the cumulative gamma histograms after optimization (Figure 3e, f).

## 4 DISCUSSION

As is well known, plan validation is crucial for radiation therapy. However, a standard CSI verification process is lacking. Most centers typically perform dose verification for CSI by dividing the target volume into three segments—cranial, upper spine, and lower spine—rather than verifying the entire craniospinal target as a single integrated volume.^7^ In our institution, the current practice for CSI dose verification employs either point dose or planar dose segmentation methods. However, these approaches still present the aforementioned limitations. To address these challenges, we propose to implement DA capable of dose reconstruction based on LOTs for single‐session DQA of CSI in HT.

Several studies^15^, ^16^, ^17^, ^18^ have reported the feasibility of using LOTs reconstructed from MVCT detector data for 3D dose verification. Han et al.^18^ verified 20 conventional plans using an independent dose reconstruction algorithm and compared them with an Exradin A1SL ionization chamber and radiochromic EBT3 film doses, using a standard of 3%/3 mm. A point dose error < 5% and a γ passing rate < 90% were considered a failure. Their research showed that differences between the planned and measured data in all except one case were within the acceptance criteria and comparable to those of conventional QA. Those researchers also assessed 100 plans using the same algorithm, showing that the point dose error and γ passing rate from HT detector data were comparable to conventional QA methods.^19^ Thiyagarajan et al.^20^ compared 3D doses reconstructed from an internal algorithm with an ionization chamber and ArcCHECK for five patients with total marrow and lymphoid irradiation. They showed that the reconstruction method resulted in poor mean ± SD 3D γ of 92.00% ± 5.83%, 64.80% ± 28.28%, 69.20% ± 30.46%, 60.80% ± 19.37%, and 73.2% ± 20.36% for PTV‐brain, ‐chest, ‐torso, ‐limb, and ‐upper body, respectively. Optimization of all upper body  total marrow irradiation/ total marrow and lymphatic irradiation plans with new pitch and modulation factors of 0.3 and 3 led to a significant improvement with 3D γ of 100% for all PTVs. While the feasibility of using LOT for 3D dose verification has been confirmed in some patients, studies on CSI are few. Deshpande et al.^12^ reported three CSI plans, focusing on the times of a dose reconstruction tool (35.0 ± 3.5 min) and film/point dose‐based measurement (90.0 ± 5.2 min). However, dose comparison was lacking. The trial used DA software with a default 6%/3 mm for 3D γ analysis, defining plan pass if the γ passing rate was ≥96%. However, further research is needed to determine whether this standard suits clinical practice.

To evaluate the reliability of DA, we performed a comparative analysis with MatriXX. The selection of MatriXX among available dosimetric tools used in our center was based on its technical advantages. While ionization chambers are limited to single‐point measurements, film dosimetry is associated with inherent uncertainties in processing and densitometry, ArcCheck is constrained by a 21 cm measurement length in the head‐to‐foot direction. In contrast, MatriXX combines a larger head‐to‐foot measurement length (24 cm) with operational efficiency and comprehensive dose acquisition capabilities, making it the optimal choice for this comparative study.

In our study, both area under the curves were greater than 0.700, indicating a high DA accuracy in CSI verification.The optimal passing rate thresholds for PTV‐cranial and PTV‐spine plans were 89.0% and 86.0%, respectively, both lower than the AAPM TG‐218 recommended passing rate threshold of 95%. The difference of γ passing rate might be because our DA study only analyzed the dose differences in PTV, while MatriXX analyzed dose differences across the entire coronal plane, and the studies cited above analyzed all voxels. For PTV‐cranial plans, the sensitivity (0.700) and specificity (1.000) at the optimal passing rate threshold were both satisfactory. The sensitivity effectively minimized the risk of misclassifying acceptable plans, thereby reducing the potential increase in the daily workload of physicists. Furthermore, the specificity ensured the accurate identification and rejection of unacceptable plans. For PTV‐spine plans, the specificity at the optimal passing rate threshold was 0.900, which maximized the prevention of unacceptable plans from being approved, aligning with clinical requirements and reducing unnecessary harm to patients due to misjudgments. However, the sensitivity at the optimal passing rate threshold was 0.524, indicating that a portion of acceptable plans were misclassified as unacceptable. These plans required further validation by physicists using additional DQA tools. Overall, the application of DA in CSI maintained DQA accuracy since misclassified plans would require follow‐up using additional DQA tools, as mentioned earlier.

As for those failed plans, we tried to analyze the risk factors for DQA failure with the results of DA and MatriXX. Turcas et al.^21^ reviewed 56 studies on HT for CSI, finding that most plans used a low MF (range, 1.5–3.0; median, 2.3). All plans met clinical requirements for dose distribution and constraints on organs at risk but did not report dose verification. Several studies^22^, ^23^, ^24^, ^25^ reported risk factors such as the percentage of LOT below 100 ms, sites, treatment time over dose per fraction and MF affecting DQA failure in HT. Binny et al.^22^ found that the range of MF‐actual used by the TPS between 1.4 and 2.5 fall within the optimal scope of passing delivery quality assurance for most plans across a range of treatment sites, the corresponding MF‐plan is between 2.0 and 3.4. This work illustrates that the use of very fast or slow LOTs can exceed the machine limitations of leaf motions, which can translate into inaccuracies between a plan and its delivery. From a previous study^24^ it was also concluded that increasing mean LOT for failed plans in small increments may improve delivery accuracy. At present, there has been little research on the reasons for DQA failure of tomo's CSI plan validation. In this study, Univariate logistic regression showed that the MF‐plan, the MF‐actual, beam on time, gantry period and Maximum LOT were independent risk factors for DA. Only the MF‐plan parameter could be adjusted when redesigning the plan. Clinically, we also observed that increasing the MF‐plan improved the plan verification passing rate. Therefore, we reoptimized 11 failed plans with MF‐plan = 2.6, resulting in nine plans passing verification and significantly improved DA and MatriXX passing rates.

We aimed to investigate the fundamental mechanisms underlying the impact of MF on passing rate. In the plan illustrated in Figure 3, the MF‐plan was set to 2.2, while the MF‐actual was 1.9, and the Mean LOT was 147.6 ms. Based on the definition of MF, the Maximum LOT was constrained to approximately 280 ms. Due to the limited beam delivery range in the spinal region and the relatively low MF‐plan value, the spike was observed around the maximum LOT, contributing to the suboptimal passing rate. AAPM Task Group Report 306^26^ demonstrated that the existence of mechanical transit time prevents the same leaf from having two transition events within a very short period of time, thus preventing the delivery of very short leaf open and closing times (on average ∼18 ms). When the commanded LOTs slightly less than 235 ms are followed by projections with commanded LOTs also around 235 ms, the mechanical transit time limitation prevents very short leaf close times from being delivered. This results in longer open projections than commanded. Spinal plans need to avoid the lungs, resulting in a higher proportion of long and short leaf openings and a lower spinal plan passing rate. This study analyzed the proportion of LOTs exceeding 235 ms, finding a negative association between MF and the proportion of long openings. Possibly for this reason, increasing the MF‐plan improved the passing rate. In summary, selecting an appropriate MF for CSI planning is important to meet clinical plan evaluation and verification.

This study has several limitations. Although the 3D doses reconstructed by DA included the target and organs at risk (OARs), the MatriXX's inability to provide comprehensive 3D dose data restricted validation for OARs. Additionally, the small sample size may limit the generalizability of the findings. Future studies should aim to include a larger number of cases to validate the consistency and accuracy of the DA across diverse clinical scenarios. Furthermore, the study focused primarily on γ analysis as the metric for dose comparison, which may not fully capture all clinically relevant dosimetric differences. Additional dosimetric parameters, such as DVH analysis for OARs, could provide deeper insights into the clinical implications of the observed discrepancies. Multicenter studies are also needed to confirm reproducibility and further evaluate the DA's clinical utility. These efforts would contribute to a more thorough understanding of the DA's performance and its potential role in improving the accuracy and safety of radiation therapy.

## 5 CONCLUSION

It is effective and feasible for DA to verify the CSI plan in HT. DA could perform DQA in CSI plans without verification phantoms and solve long‐target verification issues. The MF‐plan is a significant parameter affecting verification in HT planning. An appropriate MF‐plan is essential for plan design and DQA.

## AUTHOR CONTRIBUTIONS

Xiunan Wang and Wenjie Ni wrote the main manuscript text. Yongqing Ge and Bo Liu performed the data analysis. Qin Wang, Hui Yang, and Yizhen Jin performed the formal analysis. Linan Song and Xiaofeng Mu performed the validation. All authors gave final approval of this manuscript for submission.

## CONFLICT OF INTEREST STATEMENT

The authors declare no conflicts of interest.

## ETHICAL APPROVAL

This study was approved by the Ethics Committee of Beijing Shijitan Hospital, Capital Medical University (Approval No. IIT2025‐033‐001).
